# Supplementary material for: Predicted Environmental Risk Assessment of Antimicrobials with Increased Consumption in Portugal during the COVID-19 Pandemic; The Groundwork for the Forthcoming Water Quality Survey
Source: Antibiotics (Basel). 2023 Mar 25;12(4):652. doi: 10.3390/antibiotics12040652 (PMC10135311; doi:10.3390/antibiotics12040652)
Supplement: Supplementary file 1 [file antibiotics-12-00652-s001.zip › Table S1_24.03.23.pdf]

Table S1. Excretion rate values for the selected active substances

Pharmacoterapeutic group	Selected active substances	ATCcode	Metabolisation	Excretion rate	Ref.
**Antibiotics**	Cefazoline	J01DB04	Eliminated with the urine; microbiologically active form.	0.75	[78]
	Ceftriaxone	J01DD04	Not metabolised systemically; 50 - 60 % excreted unchanged in the urine; 40 - 50 % excreted unchanged in the bile; converted to inactive metabolites by the gut flora.	0.60	[79]
	Doxycycline	J01AA02	No significant metabolism occurs; about 35–60% excreted in urine; the remainder in faeces.	1.00	[80]
	Flucloxacillin	J01CF05	10% metabolised to penicilloic acid; excretion occurs mainly through the kidney; 65% unaltered in the urine; a small portion excreted in the bile.	0.65	[81]
	Fosfomycin	J01XX01	Excreted unchanged mainly via the kidneys; 40-50% in the urine. After oral use and to a lesser extent appers in faeces (18-28%).	0.78	[82]
	Linezolid	J01XX08	30% unaltered in the urine; 40% as metabolite B, and 10% as metabolite A.; metabolites inactives.	0.30	[83]
	Meropenem	J01DH02	50 –75 % excreted unchanged in urine; 28% recovered as beta-lactam ring-opened form – without microbiological activity.	0.75	[84]
	Metronidazole	J01XD01	Metabolised mainly by hepatic oxidation and glucuronide formation; main metabolites: metabolite I (hydroxy form) present urine with 30 - 65% activity of metronidazole, and metabolite II (acid form) representing in urine, with 5% activity of metronidazole.	0.80	[85]

	Minocycline	J01AA08	Primarily metabolised to 9-hydroxy minocycline and two different N-demethylated metabolites; predominantly eliminated through the biliary route; 9% recovered in the urine, and 20-34% recovered in the faeces.	0.43	[80, 86]
	Piperacillin/ Tazobactam	J01DI54	Piperacillin metabolised to a minor microbiologically active desmethyl metabolite; 68% as unchanged substance in urine; Tazobactam metabolised to a single metabolite microbiologically inactive; 80% renal excretion unchanged;	0.68/0.80	[87]
	Rifaximin	A07AA11	Unchanged in the faeces (97%) and urine (0.32%)	0.97	[88, 89]
	Trimethoprim	J01EE01	Metabolites - most abundant: Demethylated 3'- 4'-metabolites<br>Minor: N-oxide and benzylic metabolites<br>50% to 60% of trimethoprim excreted in the urine of which approximately 80% unchanged.	0.48	[90, 91]
	Vancomycin	A07AA	No apparent metabolism; 75-80% of the drug excreted unchanged in urine.	0.80	[92]
**Antivirals**	Aciclovir	J05AB11	No significant metabolism occurs; <15% oxidised to 9-carboxymethoxymethylguanine by alcohol dehydrogenase and aldehyde dehydrogenase and 1% 8-hydroxylated to 8-hydroxy-acyclovir by aldehyde oxidase;<br>90-92% of the drug can be excreted unchanged through glomerular filtration and tubular secretion. <2% of the drug is recovered in faeces.	0.92	[93, 94]
	Lamivudine	J05AF05	Metabolism a minor route of elimination. In man, the only known metabolite of lamivudine is the trans-sulfoxide metabolite; the majority eliminated unchanged in the urine.	1.00	[95, 96]

	Emtricitabine	J05AF09	Limited metabolism; excreted by the kidneys with complete recovery of the dose achieved in urine (86%) and faeces (14%)	1.00	[97, 98]
	Raltegravir	J05AJ01	Raltegravir and raltegravir-glucuronide, detected in urine; approximately 51% and 32 % of the dose excreted in faeces and urine, respectively;	0.83	[99, 100]
	Dolutegravir	J05AJ03	Primarily metabolised through glucuronidation, 53% of a total oral dose is excreted unchanged in the faeces; the glucuronide conjugate degraded to form the parent compound in the gut lumen; 32% percent excreted in the urine, represented by ether glucuronide.	0.85	[101, 102]
	Cobicistat	J05AR14	No significant metabolism occurs; cobicistat does not undergo glucuronidation; the administered dose excreted in feces and urine: 86.2% and 8.2%, respectively.	0.94	[65]
**Antimalarials**	Hydroxy-chloroquine	P01BA02	Active metabolite: desethylhydroxychloroquine; inactive metabolites: desethylchloroquine and bidesethylchloroquine; slow elimination predominantly through the kidneys; 23-25% excreted unchanged in the urine.	0.25	[103, 104]
	Atovaquona	P01BB51	There is no evidence that atovaquone is metabolised; predominantly (≥90%) eliminated unchanged in faeces. No metabolites identified	0.90	[47, 105]
