# Supplementary material for: Predicted Environmental Risk Assessment of Antimicrobials with Increased Consumption in Portugal during the COVID-19 Pandemic; The Groundwork for the Forthcoming Water Quality Survey
Source: Antibiotics (Basel). 2023 Mar 25;12(4):652. doi: 10.3390/antibiotics12040652 (PMC10135311; doi:10.3390/antibiotics12040652)
Supplement: Supplementary file 1 [file antibiotics-12-00652-s001.zip › Table S2_24.03.23.pdf]

Table S2. Assessment of $PNEC_{ECOtox}$ and $PNEC_{MICsub}$ for the selected substances

Active substances	Most susceptible species	EC_{10}/EC_{50}	NOEC (µg/L)	AF	$PNEC_{ECOtox}$ (µg/L)	Ref.	MIC50 (µg/L)	AF	PNEC $_{MICsub}$ (µg/L)	Ref.
					**Penicillins**					
**Flucloxacillin**	*Anabaena flos-aquae*	110	30	10	11	[106]	30	100	3	[38]
**Piperacillin**	-	NA	NA	-	NA	-	6	100	0.06	[38]
**Tazobactam**	*Anabaena flos-aquae*	NA	440	10	44	[107]	4**	-	0.25	[77]
					**Carbapenems**					
**Meropenem**	*Anabaena flos-aquaea*	NA	15	50	0.3	[108]	8	100	0.08	[38]
					**Cephalosporins**					
**Cefazolin**	*Anabaena flos-aquae*	2.4	1.5	10	0.24	[109]	32***	-	1.0	[77]
**Ceftriaxone**	*Anabaena flos-aquae*	3.31	6.1	10	0.331	[42]	2***	-	0.032	[77]
					**Sulfonamides/ Trimethoprim**					
**Trimethoprim**	*Navicula pelliculosa*	1320	1200	10	132	[106, 110]	16***	-	0.5	[77]
					**Tetracyclines**					
**Doxycycline**	*Lemna gibba* (*duckweed*)	54.0	NA	100	0.54	[111]	32***	-	2	[77]
**Minocycline**	*Microcystis*	421*	NA	100	0.421	[112]	32***	-	1.0	[77]
					**Other Antibiotics**					
**Fosfomycin**	-	NA	NA	-	NA	-	125***	-	2.0	[77]
**Linezolid**	*Synechococcus leopoliensis*	90	100	100	0.9	[106]	125***	-	8.0	[77]
**Metronidazol**	*Chlorella sp.*	2030	NA	50	40.6	[106]	16***	-	0.125	[77]
**Rifaximin**	*Anabaena sp.*	NA	0.76	10	0.076	[113]	NA	-	NA	-
**Vancomycin**	*Vibrio fischeri*	4444*	NA	100	44.4	[114]	125***	-	8.0	[77]
					**Antivirals**					
**Acyclovir**	*Raphidocelis subcapitata*	3062*	1250	50	25	[71]	NA	-	NA	-
**Emtricitabine**	*Pimephales promelas*	NA	6100	10	610	[115]	NA	-	NA	-
**Lamivudine**	*Ceriodaphnia dubia*	1345*	625	10	63	[71, 116]	NA	-	NA	-
**Dolutegravir**	*Pseudokirchneriella subcapitata*	NA	95	10	9.5	[117]	NA	-	NA	-
**Raltegravir**	*Selenastrum capricornutum*	NA	3800	10	380	[118]	NA	-	NA	-

**Cobicistat**	*Pimephales promelas*	NA	4840	10	484	[66]	NA	-	NA	-
				**Antimalarials**						
**Hydroxychloroquine**	*Daphnia magna*	173	85.8	50	3.45	[119]	NA	-	NA	-
**Atovaquone**	*Ceriodaphnia dubia*	NA	0.083	100	0.00083	[48]	NA	-	NA	-

*EC_{50}
**Tazobactam+piperacillin;
***The lowest MIC value observed for any species in the EUCAST database [3];
AF: Assessment factor; ND – Data not available;
NOEC: No-Observed Effect Concentration; $PNEC_{ECOtox}$: Predicted No-Effect Concentration (Ecotoxicological); $PNEC_{MICsub}$: Predicted No-Effect Concentration (Microbiological); Ref.: References.
